# Focal myocarditis in a young male with SARS-CoV-2 infection

**DOI:** 10.1093/omcr/omaa142

**Published:** 2021-02-15

**Authors:** Ruchika Meel, Tyral D Ramsamy, Rajiv Narsing, Michelle Wong

**Affiliations:** 1 Division of Cardiology, Department of Internal Medicine, Chris Hani Baragwanath Academic Hospital and Faculty of Health Sciences, University of the Witwatersrand, Johannesburg, South Africa; 2 Department of Internal Medicine, Chris Hani Baragwanath Academic Hospital and Faculty of Health Sciences, University of the Witwatersrand, Johannesburg, South Africa; 3 Division of Pulmonology, Department of Internal Medicine, Chris Hani Baragwanath Academic Hospital and Faculty of Health Sciences, University of the Witwatersrand, Johannesburg, South Africa

## Abstract

A 31-year- old male with no comorbidities presented with chest discomfort and shortness of breath following SARS-CoV-2 infection. Primary viral myocarditis was confirmed with the aid of cardiac biomarkers and cardiac magnetic resonance (CMR) imaging. Herein, we detail his clinical presentation, management and highlight the role of CMR in viral myocarditis.

## INTRODUCTION

The true prevalence of myocarditis due to SARS-CoV-2 infection remains unknown [1]. Since the beginning of the pandemic, few cases have been reported [2]. These included those with mild disease to fulminant myocarditis. To the best of our knowledge, this is the first case report from Sub-Saharan Africa of myocarditis diagnosed with cardiac magnetic resonance (CMR) imaging.

## CASE PRESENTATION

A 31-year-old male of Indian descent, with no known comorbidities except for a history of borderline obesity in the past presented with shortness of breath and chest discomfort post SARS-CoV-2 infection. He had tested positive for SARS-CoV-2 infection about 3 weeks prior to this (mid-June 2020) presentation. He had returned to work following 2 weeks of isolation, but had difficulty coping due to exertional dyspnoea. Pulse oximetry demonstrated oxygen desaturation to 85%, particularly whilst wearing an N95 mask. His blood pressure (122/76 mmHg) and heart rate (76 beats/min) remained normal. There were no clinically relevant findings other than a body mass index of 25.3 kg/m^2^. He had poor effort tolerance on a 6-min walk test, managing a distance of only 120 m.

**Figure 1:**
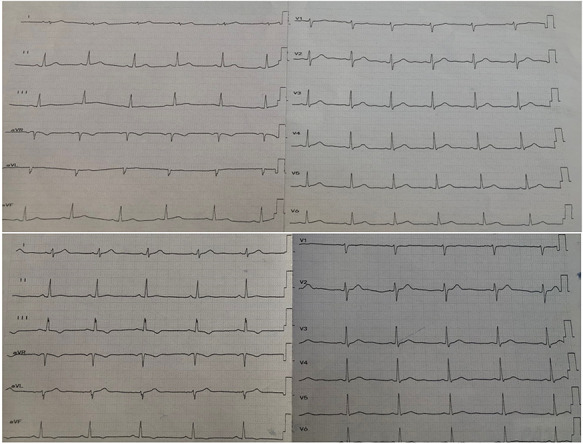
Normal baseline 12-lead ECG (top) and T wave inversion in lead III on follow-up visit (bottom).

**Figure 2:**
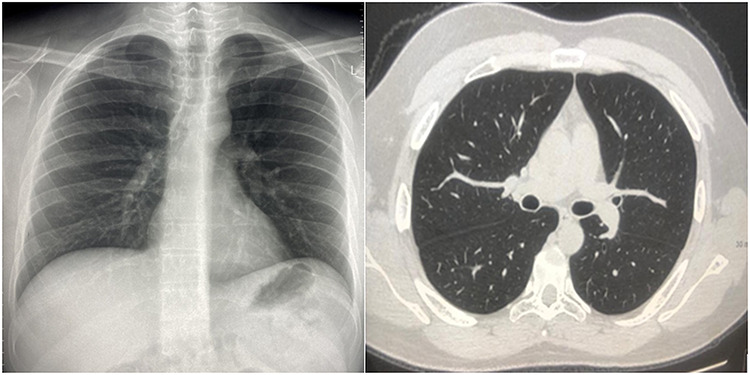
Antero-posterior view of chest radiograph (left) showing normal lung fields and CT of the chest (right) confirming absence of parenchyma lung involvement.

The following differential diagnoses were considered: pneumonia, pulmonary embolism, myocardial infarction and myocarditis. Chest X-ray and 12-lead electrocardiogram (ECG) were normal (Figs 1 and 2). Laboratory investigations showed a white blood cell count of 4.80x 10^9^/l (normal 3.92–10.4) with a normal differential count, C-reactive protein 5 mg/l (normal < 10 mg/l), ferritin 267 μg/l (normal 30–400 ug/l), D-dimer < 0.10 mg/l (0.0–0.25 mg/l) and NT-proBNP of 143 ng/l (normal < 300 ng/l). However, his high sensitivity Troponin T level was elevated at 319 ng/l (normal < 14 ng/l) and showed a steady rise to 490 ng/l. His transthoracic echocardiogram (TTE) revealed preserved left ventricular (LV) and right ventricular (RV) contractility and no regional wall motion abnormality or myocardial hypertrophy. There was no pericardial effusion. The pulmonary arteries were of normal size. Computed tomography (CT) of the chest was normal (Fig. 2). The coronary arteries were normal. Based on the clinical findings, rising troponin-T levels, and new T wave inversion in lead III on a repeat 12-lead ECG, a diagnosis of likely COVID-19-related viral myocarditis was made. Treatment with colchicine 0.5 mg twice daily and prednisone 40 mg once daily were initiated whilst awaiting a CMR scan.

**Figure 3:**
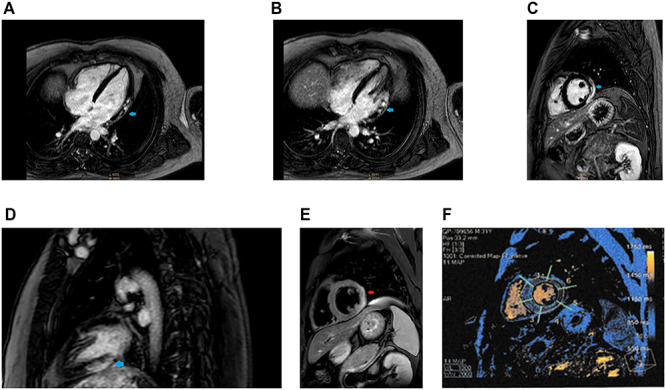
Top panels (**A** and **B**) showing LGE of the anterolateral wall (blue arrows) of the LV from the base to mid-ventricle level in the horizontal long axis view; middle panel: short- axis view of the mid-LV with LGE of the anterolateral and inferolateral wall and epicardium (blue arrow) (**C**) and LGE of the inferolateral wall of the LV (blue arrow) at the base and mid-ventricle level in the vertical long axis view (**D**); T2 STIR image showing edema of the anterolateral and infero-lateral walls (red arrow) (**E**); on non-contrast T1 mapping there was prolongation of T1 time at the mid-ventricle segment −5 and segment-6 (Image F).

The CMR scan confirmed normal LV and RV function and wall thickness with an LV ejection fraction of 65%, RV ejection fraction of 56% and wall thickness at end-diastole of 7 mm. There was delayed late gadolinium enhancement (LGE) within the mid-wall as well as the epicardial regions involving the LV basal inferolateral wall, mid-anterolateral and mid- inferolateral wall (Fig. 3A–D). T2 short tau inversion recovery (STIR) black blood imaging showed high signal intensity within the LV anterolateral, inferolateral wall at the base and mid-ventricle level (Fig. 3E). On non-contrast T1 mapping, there was prolongation of T1 time at the mid-ventricle segment 5 (1212 ± 141 ms) and segment 6 (1113 ± 93.8 milliseconds) (Fig. 3F). The aforementioned findings were compatible with Lake Louise criteria for diagnosis of focal viral myocarditis [3].

There was significant improvement in the patient’s symptoms; his 6-min walk test improved to 417 m and cardiac biomarkers returned to normal (hs-Troponin T 10 ng/l) after 1 week of medical therapy (Table 1). The treatment was continued for further 1 week. He has subsequently returned to work and is doing well.

**Table 1:** Showing initial rise and a later fall in highly sensitive troponin T (Hs-Trop T) level after institution of medical therapy on day 3 with colchicine and prednisone

Date	1 July 2020	2 July 2020	3 July 2020	6 July 2020	8 July 2020	10 July 2020	17 July 2020	4 September 2020	18 September 2020
Hs-Trop T (ng/l)	319	397	388	490	282	137	13	12	10
Medical therapy	–	–	Yes	Yes	Yes	Yes	Yes	Yes	Yes

## DISCUSSION

Myocarditis in the context of SARS-CoV-2 infection has shown great interest. The exact pathophysiology still needs to be elucidated. Currently, it is postulated that it may be the result of a cytokine storm, notably Interleukin-6, or direct invasion of the myocardium by the virus [4]. SARS-CoV-2 likely enters the myocardium by attachment to the angiotensin-converting enzyme 2 receptor on the cell surface. Pathology findings in the myocardium have been variable and non-specific [1, 5].

Myocarditis due to SARS-CoV-2 can present in the absence of pneumonia, as in this case [1, 4]. From the published reports, the clinical presentation is varied, ranging from mild symptoms such as chest pain, fatiguability and palpitations to severe disease such as ventricular dysrhythmias, sudden cardiac death and pump failure. An elevated troponin level is suggestive of myocardial involvement and implies severe SARS-CoV-2 disease [6]. However, a rise in troponin levels may be due to multiple factors in patients with SARS-CoV-2 infection. The differentials include acute coronary syndrome, pulmonary embolism, cerebrovascular accident, Takotsubo cardiomyopathy or may merely reflect demand–supply mismatch in a critically ill patient. In these patients, additional imaging is useful to exclude myocarditis as a cause of elevated troponin levels. TTE is a useful first-line bedside investigation to assess regional or global LV dysfunction, pericardial effusion, LV hypertrophy and to exclude other causes of a troponin leak. However, CMR imaging has a higher sensitivity and specificity in the diagnosis of myocarditis [1, 4]. It is particularly useful in cases where echocardiography is normal and endomyocardial biopsy (EMB) cannot be easily performed. Myocarditis typically involves the infero-postero-lateral wall at the base of the LV, but can be diffuse in nature [7]. Myocardial oedema and LGE are characteristic features of myocarditis on CMR imaging [4] as noted in the current case report. In recent reports of SARS-CoV-2-related myocarditis clinicians have not consistently made use CMR imaging or EMB for diagnosis of myocarditis [2]. The underutilization of CMR imaging and EMB makes it challenging to differentiate primary myocarditis from secondary myocardial injury.

In general, the advocated management of viral myocarditis due to SARS-CoV-2 entails use of immunosuppression, steroids, intravenous immunoglobulin and cytokine blockade [1, 4] but recommendations have varied. In addition to heart failure therapy and supportive management, steroids with or without IL-6 inhibitors, and antiviral monotherapy without immunosuppression have all been used. Our patient had localized myocarditis with mild to moderate symptoms in the absence of heart failure or respiratory involvement and was treated with a combination of prednisone and colchicine due to its inhibition of cytokines, such as interleukin-6 [8].

This case highlights and reinforces the diagnostic utility of CMR and biomarkers in SARS-CoV-2-related myocarditis.

## CONFLICTS OF INTEREST

None.

## FUNDING

None.

## ETHICAL APPROVAL

Ethics approval was obtained from University of the Witwatersrand ethics committee (M200991).

## CONSENT

Informed consent was obtained from the patient.

## GUARANTOR

Dr Ruchika Meel.
